# Acurácia do Ecocardiograma Transtorácico como Método de Triagem na Prática Clínica da Investigação da Hipertensão Pulmonar

**DOI:** 10.36660/abc.20220461

**Published:** 2023-07-27

**Authors:** Camila Farnese Rezende, Eliane Viana Mancuzo, Maria do Carmo Pereira Nunes, Ricardo Amorim Corrêa

**Affiliations:** 1 Universidade Federal de Minas Gerais Pós-Graduação Ciências Aplicadas à Saúde do Adulto Belo Horizonte MG Brasil Universidade Federal de Minas Gerais – Pós-Graduação Ciências Aplicadas à Saúde do Adulto, Belo Horizonte, MG – Brasil; 2 Universidade Federal de Minas Gerais Faculdade de Medicina Belo Horizonte MG Brasil Universidade Federal de Minas Gerais – Faculdade de Medicina, Belo Horizonte, MG – Brasil

**Keywords:** Ecocardiografia, Hipertensão Pulmonar, Confiabilidade dos Dados

## Abstract

**Fundamento:**

O ecocardiograma transtorácico (ETT) tem um papel de triagem no algoritmo diagnóstico da hipertensão pulmonar (HP). Estudos demonstraram uma discordância significativa entre as medições do ETT da pressão arterial pulmonar sistólica (PAPs) e da pressão atrial direita (PAD) e as obtidas pelo cateterismo do coração direito (CCD).

**Objetivo:**

Comparar as medições do ETT da PAPs e da PAD com as obtidas pelo CCD em pacientes com suspeita de HP.

**Métodos:**

Pacientes encaminhados a um centro de referência com probabilidade alta ou intermediária de PH ao ETT na admissão hospitalar passaram por CCD. A concordância entre a PAPs e a PAD em ambos os procedimentos foi avaliada pelo teste de Bland-Altman. Diferenças de até 10 mmHg na PAPs e de até 5 mmHg na PAD foram consideradas dentro da variabilidade do teste. A curva de característica de operação do receptor (ROC) foi construída para determinar os valores mais precisos de PAPs e VRT associados ao diagnóstico de HP pelo CCD. O nível de significância estatística adotado foi 5%.

**Resultados:**

Foram incluídos noventa e cinco pacientes. A análise de Bland-Altman análise revelou um viés de 8,03 mmHg (IC 95%: -34,9 a 50,9) na PAPs e -3,30 mmHg (IC 95%: -15,9 a 9,3) na PAD. AUC da PAPs e VRT medidas pelo ETT para a discriminação de provável HP foram de 0,936 (IC 95%: 0,836 a 1,0) e 0,919 (IC 95%: 0,837 a 1,0), respectivamente. Entretanto, apenas 33,4% da estimativa ecocardiográfica da PAPs e 55,1% da PAD foram precisas, em comparação às medições obtidas pelo CCD.

**Conclusão:**

O ETT tem um alto poder discriminatório como método diagnóstico de triagem para HP, apesar de apresentar discordâncias entre os valores absolutos de PAPs e PAD, em comparação às medições por CCD.

**Figura Central:**
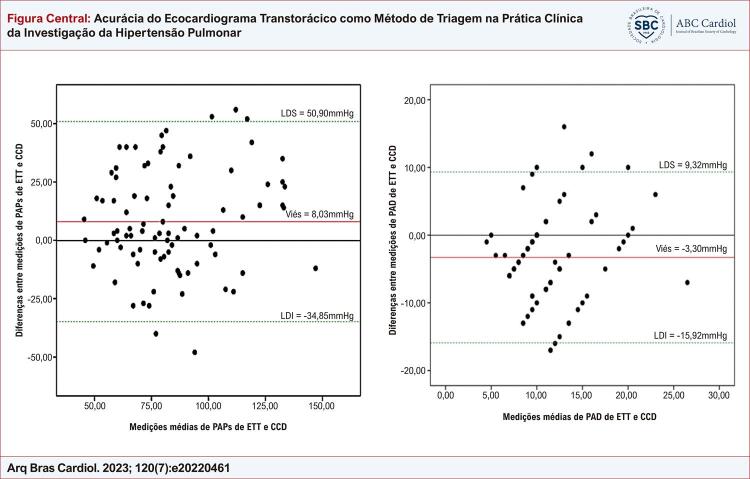
: Acurácia do Ecocardiograma Transtorácico como Método de Triagem na Prática Clínica da Investigação da Hipertensão Pulmonar

Gráfico de Bland-Altman da pressão arterial pulmonar sistólica e da pressão atrial direita medida por ecocardiograma transtorácico e cateterismo cardíaco direito.

## Introdução

A hipertensão pulmonar (HP) é uma síndrome ampla atualmente definida pela presença de pressão arterial pulmonar média (PAPm) acima de 20 mmHg, medidas pelo cateterismo do coração direito (CCD).^1^ A HP aumenta a pós-carga do ventrículo direito (VD), causando hipertrofia da camada medial da artéria pulmonar, dilatação do VD com redução da contratilidade, o que pode levar à insuficiência cardíaca direita e à morte.^2^ A HP em si é um marcador de pior prognóstico. A HP foi classificada em cinco grupos de acordo com os mecanismos patogênicos subjacentes: Grupo 1: Hipertensão arterial pulmonar (HAP); Grupo 2: HP devido a doenças cardíacas do lado esquerdo; Grupo 3: HP devido a doenças pulmonares e/ou hipóxia; Grupo 4: HP devido a obstruções arteriais pulmonares; Grupo 5: HP com mecanismos indefinidos e/ou multifatoriais.^1 - 4^ A HP é classificada ainda como pré-capilar se a PAPm for >20 mmHg, a pressão de oclusão da artéria pulmonar (POAP) for ⩽15 mmHg e a resistência vascular pulmonar (RVP) for >2 UW, incluindo os Grupos 1, 3, 4, e 5 da classificação atual.^4^ Recomenda-se a realização de um ecocardiograma transtorácico (ETT) em todos os casos com suspeita clínica de HP, já que ele tem um papel fundamental como método de triagem para o diagnóstico de HP.^3 , 4^ A velocidade máxima da regurgitação tricúspide (VRT) e a pressão arterial pulmonar sistólica (PAPs) estimada - obtida pela equação de Bernoulli modificada em que $\mathrm{PAPs}\;=\;4{\mathrm{VRT}}^{2}\;+\;\mathrm{pressão}\;\mathrm{atrial}\;\mathrm{direita}\;(\mathrm{PAD})$ – constituem parâmetros essenciais para a classificação da triagem em probabilidade de HP baixa, intermediária ou alta, sendo as últimas duas indicações para CCD confirmatório, principalmente em casos com suspeita de HAP.^3 - 5^

Entretanto, estudos realizados no contexto de protocolos de pesquisa demonstraram uma discordância significativa na medição da PAPs pelo ETT (ETT/PAPs) em comparação com a obtida pelo CCD (CCD/PAPs), devido à subestimação e à superestimação dessa variável, que pode causar atrasos no diagnóstico e no controle clínico da HP.^6 - 8^ Fora do ambiente controlado de pesquisa, em que o ETT e o CCD são realizados de forma cega e em sequência, na prática clínica usual para o diagnóstico da HP, a grandeza e a direção dessa discordância não foram relatadas. Nesse contexto, foram usadas avaliações observacionais e pragmáticas para determinar a precisão de métodos diferentes para avaliação diagnóstica da HP, com o objetivo de obter dados mais representativos em uma população não selecionada de forma não estrita.^9 , 10^

O objetivo do presente estudo foi avaliar a concordância entre ETT/PAPs e CCD/PAPs, ETT/PAD e pressão atrial direita no CCD (CCD/PAD) dos pacientes que foram encaminhados para avaliação diagnóstica em um centro de referência de HP no Brasil, na condição de prática clínica diária antes do CCD e do início de terapias medicamentosas específicas para HAP.

## Métodos

### População do estudo

Este estudo cadastrou pacientes consecutivos que haviam sido encaminhados para avaliação diagnóstica no Departamento de Doença Vascular Pulmonar do Hospital das Clínicas da Universidade Federal de Minas Gerais (DVP-HC/UFMG), em Belo Horizonte, Minas Gerais, de setembro de 2004 a abril de 2015.

Os participantes que se qualificam eram os com 18 anos de idade ou mais, que tinham suspeita clínica e laboratorial de hipertensão arterial pulmonar (HAP) do Grupo 1, ou hipertensão pulmonar tromboembólica crônica (HPTEC) do Grupo 4, com probabilidade de HP alta ou intermediária de acordo com o ETT, e que passaram por CCD para confirmar o diagnóstico.^3 , 4^ Um intervalo máximo de até 6 meses entre as datas do ETT e o CCD foi aceitável nesse contexto.^8^

Pacientes com diagnóstico de HP devido a doença cardíaca do lado esquerdo (HP do Grupo 2), associada a doenças pulmonares e/ou hipóxia (HP do Grupo 3) ou com mecanismos não claros e/ou multifatoriais (HP do Grupo 5), não se qualificaram para o estudo, já que o CCD não é formalmente indicado para o diagnóstico desses grupos de HP.^1 , 3 , 4^

Este estudo foi aprovado pelo Comitê de Ética da UFMG (ETIC nº 1.057.219/2015), e todos os participantes que concordaram em participar assinaram o Termo de consentimento livre e informado.

### Ecocardiografia e cateterismo do coração direito

O ETT e o CCD foram solicitados pelos médicos assistentes no Centro de Referência em HP, de acordo com o protocolo local e foram realizados regularmente conforme a prática clínica da unidade de cardiologia do HC/UFMG.

A avaliação pelo ETT incluiu as variáveis VRT, PAD e PAPs, sendo esta última estimada pela fórmula modificada da equação de Bernoulli $\left(PAPs=4{VRT}^{2}+PAD\right)$ . Outros parâmetros de ETT que sugeriam HP e medições da função do VD incluíram a excursão sistólica do plano anular tricúspide (TAPSE) e a variação fracional da área do VD (FAC).^3 - 5 , 11^ A PAD foi estimada pela manobra inspiratória conforme apresentado: 3 mmHg (0 a 5 mmHg) se o diâmetro da veia cava inferior (VCI) fosse menor que 2,1 cm e diminuísse mais de 50%; 15 mmHg (10 a 20 mmHg) se o diâmetro da VCI fosse maior que 2,1 cm e diminuísse menos de 50%. Quando os critérios não eram atendidos, foi usado um valor intermediário de 8 mmHg (5 a 10 mmHg).^5 , 11^ Outros parâmetros de ETT que sugeriram HP foram a presença de pelo menos duas das três categorias de alterações: a) em relação aos ventrículos: razão do diâmetro basal do ventrículo direito/ventrículo esquerdo >1 e/ou achatamento do septo interventricular e razão TAPSE/PAPs <0,55 mm/mmHg, representando uma medição não invasiva de acoplamento arterial pulmonar do VD; b) artéria pulmonar: tempo de aceleração na artéria pulmonar <105 ms, e/ou aumento da velocidade de regurgitação pulmonar >2,2 m/s, e/ou aumento do diâmetro da artéria pulmonar >25 mm; c) VCI e átrio direito: VCI maior que 2,1 cm associado à sua redução <50% e/ou área do átrio direito >18 cm^2^ .^4 , 5 , 11^

O ETT foi classificado em três categorias de probabilidade pré-CCD de HP: a) alta probabilidade, se a VRT fosse superior a 3,4m/s ou entre 2,9 e 3,4m/s se estivesse associada a outros sinais ecocardiográficos (como acima); probabilidade intermediária, se a VRT estivesse entre 2,9 e 3,4m/s sem outros sinais de ETT, ou se a VTR fosse igual ou inferior a 2,8m/s associada a pelo menos um sinal adicional de ETT sugestivo de HP; e baixa probabilidade se nenhuma dessas variáveis estivesse presente.^3 , 4 , 11^ Esses dados foram obtidos a partir do primeiro ETT realizado no DVP-HC/UFMG, utilizando equipamentos disponíveis comercialmente (iE33, Epiq 7, Philips Medical Systems e Aplio 300, Toshiba Ultrasound Systems) e seguindo diretrizes padronizadas.^3 - 5 , 11^ A ecocardiografia foi realizada na prática de rotina por quatro ecocardiógrafos diferentes que oferecem uma avaliação abrangente da função do VD e estimativa da pressão arterial pulmonar. Todos os relatórios ecocardiográficos seguiram um protocolo padronizado para avaliar pacientes com hipertensão pulmonar.

O CCD foi realizado na Unidade Cardiovascular do HC-UFMG, por dois profissionais com extensa experiência em exames. O diagnóstico de HAP (Grupo 1) foi confirmado se houvesse PAPm igual ou superior a 25 mmHg em repouso e pressão de oclusão da artéria pulmonar (POAP), pressão do átrio esquerdo ou pressão diastólica final do ventrículo esquerdo igual ou inferior a 15 mmHg (HP pré-capilar) e resistência vascular pulmonar de 3 unidades Woods ou mais, conforme definido pelas diretrizes internacionais vigentes à época dos exames.^3^ Além disso, os pacientes com HAP tiveram que ter estudos negativos para tromboembolismo pulmonar crônico (HP do Grupo 4 – uma cintilografia pulmonar de ventilação/perfusão normal ou de baixa probabilidade ou angiografia pulmonar por tomografia computadorizada (APTC) negativa) e para doenças cardíacas do lado esquerdo (HP do Grupo 2), doenças pulmonares ou hipóxia crônica (HP do Grupo 3) e HP relacionada a outras condições diversas (HP do Grupo 5). O diagnóstico da hipertensão pulmonar tromboembólica crônica (HPTEC) do Grupo 4 foi estabelecido pela presença de HP pré-capilar assim como para HAP, mas associada a cintilografia pulmonar de ventilação/perfusão de alta probabilidade ou uma APTC positiva. No CCD, a PAD, a PAPs, a PAPm e a POAP foram registradas ao final de uma expiração normal. O débito cardíaco (DC, L/min^1^) foi calculado com base no método indireto de Fick, que estima o consumo de oxigênio (VO_2_, ml/min); o índice cardíaco (IC, L·min^1^·m^2^) foi calculado como a razão de DC para área de superfície corporal. A RVP foi calculada pela seguinte fórmula: RVP = (PAPm-POAP)/DC. Todas as medições foram obtidas com referência ao nível zero na linha torácica média.^12^

### Análise estatística

A distribuição de dados foi verificada pelo teste de Shapiro-Wilk. As estatísticas descritivas foram apresentadas como frequência e porcentagem, média (desvio padrão) ou mediana (intervalo interquartil), conforme indicado. As concordâncias entre ETT/PAPs e CCD/PAPs, e ETT/PAD e CCD/PAD, foram analisadas pelo método de Bland e Altman, juntamente com os coeficientes de variação (CV) e de repetição (CR).^13 - 15^ A estimativa do viés (diferenças médias nas medições de ETT/PAPs e CCD/PAPs, e ETT/PAD e CCD/PAD), seu desvio padrão (DP) e o limite de concordância 95% de concordância foram calculados para a preparação do gráfico de Bland-Altman.^13 , 14^ Esse método avalia o erro de medição, calculado dividindo o desvio padrão das diferenças médias pela raiz quadrada de dois.^15^ O CV é uma medição de dispersão que descreve a quantidade de variabilidade de dados relacionada à média, que foi calculada usando a seguinte fórmula: CV = DP da diferença média das medições feitas pelo ETT e pelo CCD *sobre* a média das médias dessas medições multiplicada por 100.^14^ O CR expressa a variação esperada dos resultados para 95% das medições repetidas, e é calculado da seguinte maneira: CR= DP da diferença média das medições feitas pelo ETT e pelo CCD multiplicado por 1,96.^13^ Diferenças de 5 mmHg na PAD e de 10 mmHg na PAPs entre o ETT e o CCD foram consideradas clinicamente aceitáveis.^6^ A curva de característica de operação do receptor (ROC) foi construída para determinar os valores mais precisos de PAPs e VRT associados ao diagnóstico de HP pelo CCD na presente coorte e para verificar a precisão do valor de ETT/PAPs >36 mmHg e de VRT ≥ 2,80m/s, os valores de corte recomendados anteriormente pela literatura para fazer a triagem de pacientes com sintomas de HP pelo ETT.^16 , 17^

O poder da amostra foi calculado usando o teste t pareado para permitir a avaliação da concordância das medidas PAPs entre o ETT e o CCD, usando o pacote estatístico Minitab Release 14. Para um poder estatístico de 80% na estimativa da diferença real nas medidas de PAPs entre o ETT e o CCD, considerando uma diferença clinicamente aceitável de 10mmHg e um nível alfa de 0,05, estimou-se um tamanho amostral de 90 pacientes. Um p-valor abaixo de 0,05 foi considerado significativo para todas as outras análises. O Statistical Package for the Social Sciences, versão 18, foi utilizado para as análises.

## Resultados

Noventa e cinco pacientes foram admitidos consecutivamente no DVP-HC/UFMG e atenderam aos critérios de inclusão no período de estudo. Um paciente foi excluído porque não foi possível recuperar as medições de ETT/PAPs e ETT/PAD antes do CCD. Cinco pacientes não tiveram diagnóstico confirmado de HP pelo CCD. A coorte foi composta de participantes de meia-idade, cuja maioria era de mulheres nas classes funcionais II e III. Aproximadamente dois terços da amostra era composta de pacientes com HAP, e os demais eram pacientes com HPTEC. Nenhum paciente estava em tratamento farmacológico para a HAP no momento dos exames ( Tabela 1 ).

**Tabela 1:** – Características demográficas e clínicas de linha de base da população do estudo (n=95)

Características	Dados*
**Idade, média ± DP, (anos)**	47,6 ± 14,5
**Feminino, n (%)**	66 (69,4%)
**FC NYHA, n (%)**	
I	8 (8,4%)
II	37 (38,9%)
III	40 (42,1%)
IV	10 (10,5%)
**Prevalência de HP**	
Sem HP, n (%)	5 (5,2%)
HP, n (%)	90 (94,8%)
**Intervalo entre medições não invasivas e invasivas, mediana (IIQ), dias**	104 (62-153)
**HAP, n (%)**	56 (62,9%)
Esquistossomose	19 (33,9%)
Idiopática	14 (25%)
Doença cardíaca congênita	9 (16,1%)
Doença de tecido conjuntivo	8 (14,8%)
Hipertensão portopulmonar	4 (7,1%)
HIV	2 (3,6%)
**HPTEC – n (%)**	33 (37,1%)

*Os dados são apresentados como média ± DP ou mediana (IIQ: intervalo interquartil). HPTEC: hipertensão pulmonar tromboembólica crônica; CF: classe funcional (New York Heart Association); HIV: vírus da imunodeficiência humana; HAP: hipertensão arterial pulmonar; HP: hipertensão pulmonar.

Os dados do ETT e do CCD estão descritos na Tabela 2 .

**Tabela 2:** – ETT e parâmetros hemodinâmicos da população do estudo (n=95)

Exames	Variáveis medidas	Valor
ETT	PAPs, média ± DP, mmHg*	79,9 ± 24,7
	PAD, média ± DP, mmHg	12,9 ± 4,7
	VRT, média ± DP, m/s*	3,78 ± 0,71
	TAPSE, mediana (IIQ), mm	16 (15-18)
	VD-FAC, mediana (IIQ), %	32 (24-38)
	Razão TAPSE/PAPs, mediana (IIQ), mm/mmHg	0,28 (0,19-0,39)
CCD	PAPs, média ± DP, mmHg	87,6 ± 27,2
	PAPm, média ± DP, mmHg	70,2 ± 14,4
	PAD, média ± DP, mmHg	9,6 ± 5,6
	POAP, mediana (IIQ), mmHg	10,0 (9,2 - 12,4)
	IC, mediana (IIQ), L.min^−1^.m^−2^	2,46 (1,71 - 3,36)
	RVP, média (IIQ), unidades Wood	6,6 (5,1 - 8,2)

Os dados são apresentados como média ± DP ou mediana (IIQ: intervalo interquartil). IC: índice cardíaco; PAPm: pressão arterial pulmonar média; POAP: pressão de oclusão da artéria pulmonar; RVP: resistência vascular pulmonar; PAPs: pressão arterial pulmonar sistólica; PAD: pressão atrial direita; CCD: cateterismo do coração direito;VD-FAC: variação fracional da área do ventrículo direito; TAPSE: excursão sistólica do plano anular tricúspide; VRT: velocidade de regurgitação tricúspide; ETT: ecocardiograma transtorácico. *Um paciente não apresentava regurgitação tricúspide e foi excluído na análise do ETT PAPs e VRT.

**Figura 1:**
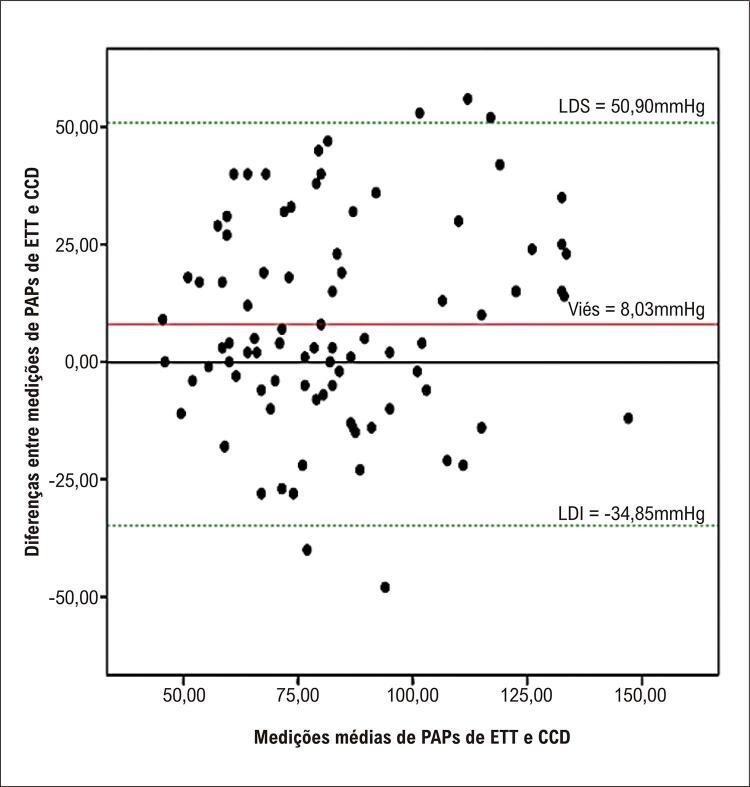
– Gráfico de Bland-Altman da pressão arterial pulmonar sistólica medida por ecocardiograma transtorácico e cateterismo do coração direito. *LDI: limite de discordância inferior; PAPs: pressão arterial pulmonar sistólica; CCD: cateterismo do coração direito; ETT: ecocardiograma transtorácico; LDS: limite de discordância superior.

Um viés estatisticamente significativo foi determinado entre as medições de ETT/PAPs e CCD/PAPs, conforme apresentado na Figura 1 e na Ilustração central. Em relação à concordância entre os parâmetros ( Tabela 3 ), o erro de medição foi de 15,5 mmHg, o CV foi de 26% e o CR foi de 42,9 mmHg.

**Tabela 3:** – Acurácia do ETT, PAPs, e VRT na predição da hipertensão pulmonar*

Variável	Valor	Sensibilidade	Especificidade	VPP	VPN
ETT/PAPs	48 mmHg^1^	90,7%	100%	100%	28,6%
	36 mmHg^2^	97,7%	2,1%	95%	4,6%
VRT	3,08 m/s^1^	90,7%	100%	100%	33,3%
	2,80 m/s^2^	95,3%	5,4%	95%	5,7%

PAPs: pressão arterial pulmonar sistólica; VRT: velocidade de regurgitação tricúspide; ETT: ecocardiograma transtorácico; VPP: Valor preditivo positivo; VPN: Valor preditivo negativo. *PAPs medida pelo cateterismo do coração direito como a referência. Para o VRT, a referência foi o achado da definição de HP pelo CCD (consulte as definições no texto). 1- Valores de ETT/PAPs e VRT encontrados na coorte. 2- Valores de ETT/PAPs e VRT definidos pela literatura.

**Figura 2:**
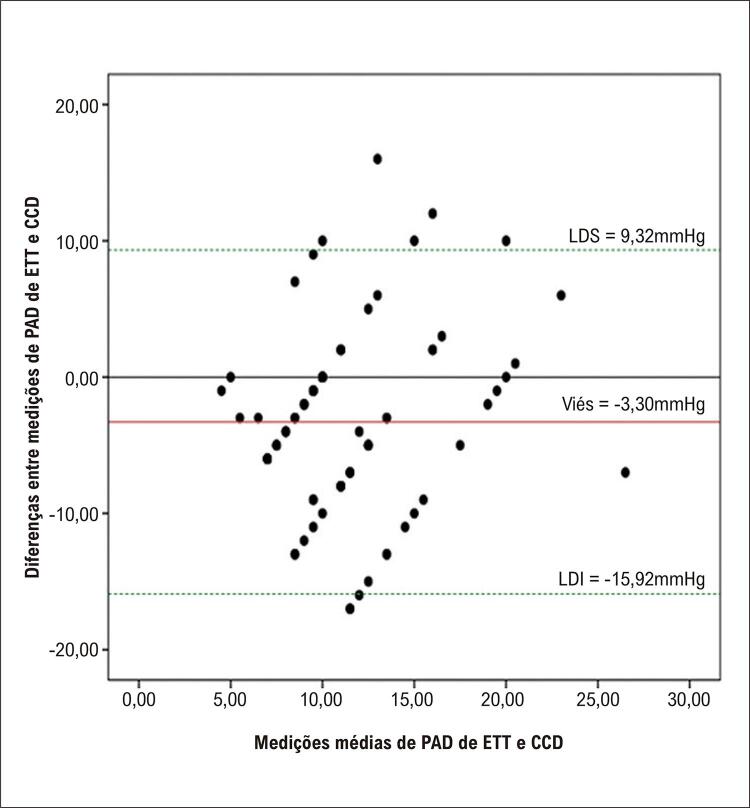
– Gráfico de Bland-Altman da pressão atrial direita medida por ecocardiograma transtorácico e cateterismo do coração direito. *LDI: limite de discordância inferior; PAD: pressão atrial direita; CCD: cateterismo do coração direito; ETT: ecocardiograma transtorácico; LDS: limite de discordância superior.

A Figura 2 e a Ilustração central mostram a concordância entre o ETT/PAD e o CCD/PAD. Um viés significativo foi encontrado entre as medições.

**Figura 3:**
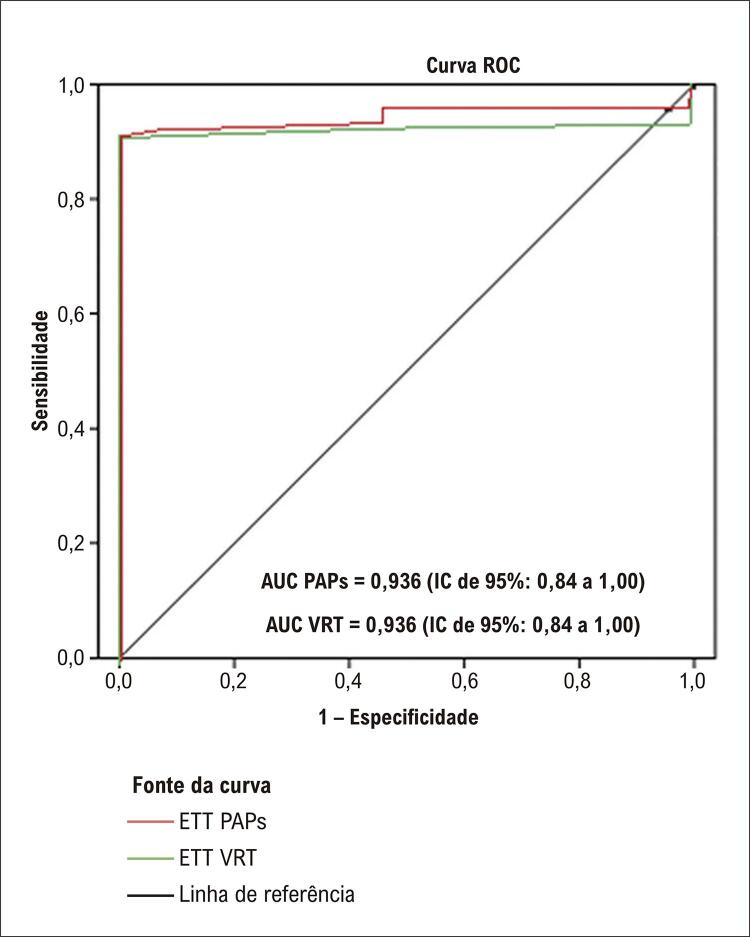
– A AUC da PAPs e VRT mais precisas medidas por ETT para prever a hipertensão pulmonar na coorte. * AUC: área sob a curva; VPN: valor preditivo negativo; VPP: valor preditivo positivo; PAPs: pressão arterial pulmonar sistólica; VRT: velocidade de regurgitação tricúspide; ETT: ecocardiograma transtorácico.

Na presente coorte, quanto mais altos os valores de ETT/PAPs e VRT, mais alto o poder discriminatório para o diagnóstico de HP pelo CCD ( Figura 3 e Tabela 3 ). Por outro lado, os valores de PAPs e VRT anteriormente recomendados como pontos de corte da probabilidade intermediária de HAP pelo ETT apresentaram sensibilidades mais altas em detrimento de especificidades mais baixas.^16^

**Figura 4:**
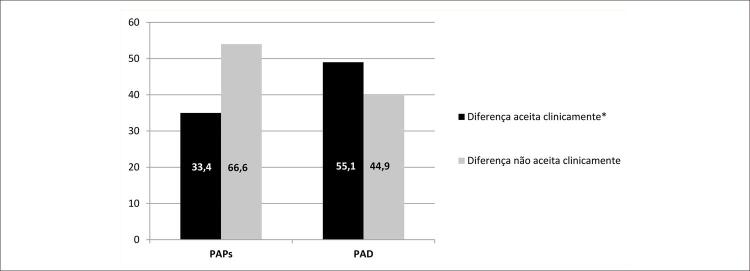
– Diferenças clinicamente aceitáveis para PAPs e PAD entre o ecocardiograma transtorácico e o cateterismo do coração direito. PAPs: pressão arterial pulmonar sistólica; PAD: pressão atrial direita. Diferenças de 5 mmHg na PAD e de 10 mmHg na PAPs entre o ecocardiograma transtorácico e o cateterismo do coração direito foram consideradas clinicamente aceitáveis.

**Figura 5:**
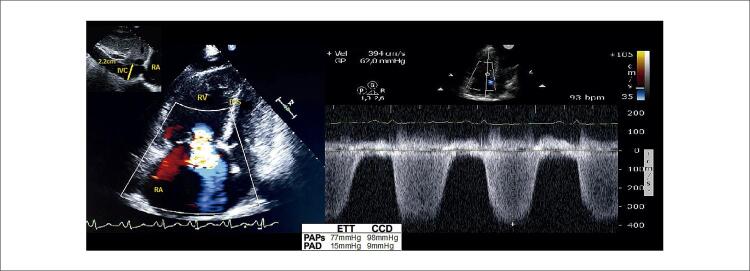
– Discordância entre a pressão arterial pulmonar e a pressão atrial direita medida por meio de ETT e CCD em paciente com hipertensão pulmonar associada à esquistossomose. VCI: veia cava inferior; SIV: septo interventricular; AD: átrio direito; CCD: cateterismo do coração direito; VD: ventrículo direito; PAPs: pressão arterial pulmonar sistólica; ETT: ecocardiograma transtorácico.

Pela definição de acurácia adotada para PAPs (variação de até 10mmHg) e PAD (variação de até 5mmHg) no ETT em comparação com as do CCD, as estimativas de PAD e PAPs foram ruins ( Figura 4 ). O ETT subestimou os valores de PAPs em 41,5% versus 25,1% (-30,4±10,2 versus 15,2±8,9 mmHg; p=0,04) e superestimou os valores de PAD em 33,7% versus 11,2% (11,3±4,8 versus -8,4±3,7 mmHg; p=0,03) nos casos em que a diferença estava acima da variação aceitável pré-definida. Ilustração da avaliação do ETT da PAPs e da PAD em um paciente com esquistossomose associada à HAP e a diferença dessas medições no CCD são apresentadas na Figura 5 .

## Discussão

O presente estudo de centro único buscou avaliar a acurácia do ETT na triagem de HP em pacientes encaminhados a um centro de referência de HP no contexto de prática clínica diária. O ETT desempenha um papel fundamental no cenário clínico do diagnóstico da HP.^3 , 4^ Com dados clínicos e laboratoriais, a probabilidade de HP pelo ETT direciona a investigação para um dos cinco grupos atuais da doença, que exigem outros procedimentos e terapias específicos.^3 , 4 , 18^ Pacientes com probabilidade intermediária ou alta de HP pelo ETT e com suspeita de HAP (HP do Grupo 1) ou HPTEC (HP do Grupo 4) são encaminhados primariamente para diagnóstico invasivo por CCD, pois são candidatos ao uso de modalidades terapêuticas específicas, como tratamento farmacológico para HAP ou tromboendarterectomia pulmonar combinada ou não com angioplastia pulmonar e/ou terapia medicamentosa para HPTEC.^19 , 20^

O presente estudo demonstra que tanto a ETT/PAPs maior que 36 mmHg quanto a VRT igual ou superior a 2,80m/s apresentam alta sensibilidade na triagem de HP em pacientes sintomáticos, o que é um desempenho desejável quando um teste é usado para triar o diagnóstico de doenças graves.^21^

A amostra incluída neste estudo compreendeu todos os pacientes encaminhados ao DVP-HC/UFMG que foram admitidos consecutivamente durante o período do estudo e foi composta de um número maior de pacientes com HAP (62,9%) do que com HPTEC (37,1%). Devido ao viés de encaminhamento, o número de pacientes cujo CCD foi negativo para HP foi muito baixo (5,2%). Diferentemente de relatórios de países do hemisfério norte, o *Schistosomiasis mansoni* é nossa etiologia de HAP mais prevalente devido à endemicidade dessa infecção, especialmente em nossa região.^22 - 24^ No entanto, a distribuição média de idade e sexo foi semelhante à de relatos de países do hemisfério norte, demonstrando o impacto da doença em indivíduos de meia-idade mesmo em contextos epidemiológicos diversos.^25 , 26^ Da mesma forma, a maioria dos pacientes, na admissão, estava nas classes funcionais II e IV (52,6%). Infelizmente, o atraso no diagnóstico de HP é um problema mundial que contribui para o agravamento da doença para estágios avançados, incluindo insuficiência cardíaca direita e alto risco de morte.^25 , 26^

### Comparação entre medições ecocardiográficas e hemodinâmicas invasivas das pressões da câmara direita do coração

A acurácia do ETT no diagnóstico de HP tem sido avaliada desde a década de 1980. O ETT atualmente é um procedimento de custo relativamente baixo, amplamente disponível e não invasivo. Yock e Popp relataram uma boa correlação entre ETT/PAPs e CCD/PAPs em 54 pacientes (r=0,93, SEE = 8 mmHg).^27^

No entanto, há alguma discussão sobre a adequação do uso de testes de correlação comuns quando dois métodos distintos avaliam a mesma variável dependente quantitativa.^13^ O método de Bland-Altman é considerado mais adequado para esse fim. Achados de Fisher et al. mostraram um viés significativo do ETT na estimativa da PAPs (-0,6 mmHg; limite de concordância de 95%: -40,0 a 38,8 mmHg) em 65 pacientes submetidos a ETT e CCD de forma cega e com intervalo de uma hora entre os testes.^6^ Dois outros estudos também relataram um viés que variou de 2,2 a 8mmHg na estimativa da PAPs pelo ETT (limite de concordância de 95%: -34,2 a 38,6 mmHg e -28,4 a 44,4 mmHg).^7 , 8^ A análise dos dados do registro REVEAL mostrou baixa acurácia do ETT pré-CCD em 57,4% das estimativas de PAPs (> 10 mmHg maior ou menor que a medida do CCD) e em 36,5% das estimativas de PAD (>5 mmHg maior ou menor que PAD em CCD) em exames feitos com intervalo maior que o nosso, intervalo máximo de 12 meses entre os exames.^8^

Entretanto, outros autores demonstraram uma melhor concordância entre as medidas de PAPs, mas o nível de concordância na diferença da média foi maior, indicando apenas uma precisão moderada das medições ecocardiográficas.^28 - 30^ Embora D’Alto M et al. não tenham encontrado viés significativo (-0,5 mmHg) entre ETT/PAPs e CCD/PAPs com amplos limites de concordância (-19 mmHg a 18 mmHg), ao contrário de outros relatos, o CCD foi recomendado por outros motivos que não HAP (HAP: 36%; hipertensão venosa pulmonar: 40%, doença pulmonar HP: 16%).^31^ Um grande estudo retrospectivo (n=1.695) incluiu pacientes com intervalo máximo de cinco dias entre ETT e CCD. As principais indicações foram cardiopatia esquerda (59%), valvopatia (27%) e HAP (6%). Eles encontraram uma média de PAPs de 45,3 ± 15,5 mmHg pelo ETT e 47,4 ± 16,4 mmHg pelo CCD, mostrando uma forte correlação entre as medidas de PAPs (r = 0,87; p<0,0001) e PAD (r = 0,82; p<0,0001).^32^ Pela análise de Bland-Altman, houve um viés de -2 mmHg para PAPs (95% limite de concordância: -18,1 a 14,1 mmHg) e um viés de 1 mmHg para PAD (95% de limite de concordância: 0,1 a 1,9 mmHg).^32^ Doutreleau et al. compararam ETT e CCD, que foram realizados sequencialmente em 106 pacientes com suspeita ou confirmação de HP (intervalo médio de 16 min). A HP não foi confirmada em 16,9% dos pacientes, 10,4% foram diagnosticados com HP pós-capilar e 72,7% com HP pré-capilar. As correlações foram fortes (para PAPs: r=0,84; para PAD: r=0,70), e a análise de Bland-Altman mostrou um viés significativo de 1,4 mmHg para PAPs (limite de concordância de 95%: -22,6 a 25,4 mmHg) e 1,9 mmHg para PAD (limite de concordância de 95%: -6,1 a 9,9 mmHg).^33^ Outros autores avaliaram pacientes consecutivos e recomendaram CCD (HAP e HPTEC: 40%; insuficiência cardíaca: 42%), utilizando um intervalo maior de até três horas entre os testes, e relataram um viés mínimo (viés médio= +2,4 mmHg) entre as medidas de ETT e PAPs invasiva, mas com um amplo limite de concordância (-20 a +25 mmHg).^34^

Três metanálises e uma revisão sistemática avaliaram a precisão da estimativa da PAPs pelo ETT, mas, no geral, os resultados foram inconsistentes devido a uma significativa heterogeneidade entre os estudos quanto à inclusão de participantes sem doença e outros com cardiopatia esquerda e direita. Além disso, a maioria dos estudos empregou os testes usuais de correlação, menos apropriados como medidas de concordância entre testes que medem a mesma variável quantitativa.^35 - 37^

Diretrizes internacionais publicadas recentemente atualizaram a definição hemodinâmica de HP diminuindo o valor de PAPm de ≥ 25 mmHg para > 20 mmHg.^4^ Esse ajuste foi baseado nos valores relatados anteriormente encontrados em indivíduos sem HP e no crescente corpo de evidências do impacto prognóstico de elevações leves de PAPm (entre 20 e 24 mmHg) em alguns subgrupos de HAP.^4^ Entretanto, o ETT continua a ser o método de triagem mais apropriado para o diagnóstico de HP. Nesse novo cenário, Gall et al. avaliaram uma grande amostra retrospectiva de pacientes com HP confirmada e descobriram que o gradiente de regurgitação tricúspide atende aos novos critérios de definição de HP e que diminuir o limite inferior da VRT não melhora o rendimento da triagem do ETT.^38^

Até onde sabemos, este é o primeiro estudo brasileiro publicado que comparou as pressões obtidas pelo ETT e pelo CCD em pacientes adultos com suspeita diagnóstica de HAP e HPTEC no contexto da prática clínica diária. O presente estudo demonstrou o alto poder discriminatório da ETT/PAPs e da VRT para o diagnóstico de HP. De acordo com nossas definições (diferença de até 10 mmHg para PAPs e de até 5 mmHg para PAD), apenas 33,4% das estimativas de ETT/PAPs e 55,1% de ETT/PAD foram precisas, o que foi semelhante aos resultados encontrados por Fisher et al.^6^ (PAPs: 52%), Rich et al.^7^ (PAPs: 49,4%), e análise REVEAL^8^ (PAPs: 42,6% e PAD: 63,5%). Doutreleau et al.^33^ e Venkateshvaran et al.^34^ encontraram medidas um pouco mais confiáveis de PAPs (68% e 62%, respectivamente) e PAD em 79% dos pacientes.^34^ Em relação à direção das discordâncias, o presente estudo mostrou que o ETT subestimou a PAPs e superestimou a PAD com maior frequência (PAPs: 41,5% e 25,1%; PAD: 33,7% e 11,2%) e na magnitude das diferenças absolutas (PAPs: -30,4±10,2 versus 15,2±8,9 mmHg; PAD: 11,3±4,8 versus -8,4±3,7 mmHg), todas superiores às diferenças aceitáveis pré-definidas de 10 mmHg e 5 mmHg, respectivamente. Fischer et al. observaram que os valores de PAPs foram mais subestimados do que superestimados (-30±16 versus 19±11 mmHg; p=0,03).^6^ Rich et al.^7^ e Farber et al.^8^ também encontraram dados semelhantes usando o registro REVEAL (PAPs: subestimada em 30% e 20,6% no ETT, e em 34,8% e 22,5%, respectivamente; PAD: superestimada em 26,3% versus 12,4%).^8^ Esse cenário ilustra a dificuldade de se determinar a gravidade da HP e de estratificar seu risco usando apenas o ETT.

Algumas limitações deste estudo devem ser mencionadas. Primeiramente, o tempo decorrido entre o ETT e o CCD pode levantar algumas preocupações. Entretanto, devido ao desenho observacional e aos objetivos deste estudo, o ETT e o CCD não puderam ser realizados em um intervalo curto. Além disso, encontramos um intervalo médio de 3,3 meses, o que é aceitável quando pacientes com suspeita de HP são encaminhados por não especialistas para avaliação em centros de referência em HP no contexto da prática clínica em ambiente de saúde pública. Relatórios do Reveal Registry - um registro histórico de HP – analisaram esse tópico em intervalos ainda maiores.^7 , 8^ Em segundo lugar, pode-se esperar alguma variabilidade entre os examinadores, uma vez que o ETT que levantou a suspeita de HP foi realizado por examinadores diferentes. No entanto, todos os exames foram realizados na mesma unidade cardiológica do HC/UFMG e agendados regularmente no âmbito do atendimento médico habitual. Por outro lado, este estudo teve o propósito de avaliar a consistência do ETT como triagem para HP fora do ambiente controlado de pesquisa. Nesse sentido, esperávamos discordâncias ainda maiores do que as relatadas na literatura. Isto posto, consideramos os presentes resultados altamente consistentes, pois reproduzem relatos anteriores e refletem, ao menos em parte, limitações inerentes aos métodos, independentemente dos contextos geográficos em que são realizados.^5 , 11 , 37^

## Conclusão

Em conclusão, o ETT desempenha um papel fundamental na avaliação de triagem de doentes com suspeita de HP e tem um alto poder discriminatório mesmo no contexto da prática clínica habitual. As discordâncias entre as medidas de PAPs e PAD reforçam o ETT como uma ferramenta de triagem válida e a necessidade de realizar CCD no contexto em que se recomenda um diagnóstico definitivo de HP.

## References

[1] Simonneau G, Montani D, Celermajer DS, Denton CP, Gatzoulis MA, Krowka M (2019). Haemodynamic Definitions and Updated Clinical Classification of Pulmonary Hypertension. Eur Respir J.

[2] Noordegraaf AV, Chin KM, Haddad F, Hassoun PM, Hemnes AR, Hopkins SR (2019). Pathophysiology of the Right Ventricle and of the Pulmonary Circulation in Pulmonary Hypertension: An Update. Eur Respir J.

[3] Galiè N, Humbert M, Vachiery JL, Gibbs S, Lang I, Torbicki A (2015). 2015 ESC/ERS Guidelines for the Diagnosis and Treatment of Pulmonary Hypertension: The Joint Task Force for the Diagnosis and Treatment of Pulmonary Hypertension of the European Society of Cardiology (ESC) and the European Respiratory Society (ERS): Endorsed by: Association for European Paediatric and Congenital Cardiology (AEPC), International Society for Heart and Lung Transplantation (ISHLT). Eur Respir J.

[4] Humbert M, Kovacs G, Hoeper MM, Badagliacca R, Berger RMF, Brida M (2022). 2022 ESC/ERS Guidelines for the Diagnosis and Treatment of Pulmonary Hypertension. Eur Heart J.

[5] Rudski LG, Lai WW, Afilalo J, Hua L, Handschumacher MD, Chandrasekaran K (2010). Guidelines for the Echocardiographic Assessment of the Right Heart in Adults: A Report from the American Society of Echocardiography Endorsed by the European Association of Echocardiography, a Registered Branch of the European Society of Cardiology, and the Canadian Society of Echocardiography. J Am Soc Echocardiogr.

[6] Fisher MR, Forfia PR, Chamera E, Housten-Harris T, Champion HC, Girgis RE (2009). Accuracy of Doppler Echocardiography in the Hemodynamic Assessment of Pulmonary Hypertension. Am J Respir Crit Care Med.

[7] Rich JD, Shah SJ, Swamy RS, Kamp A, Rich S (2011). Inaccuracy of Doppler Echocardiographic Estimates of Pulmonary Artery Pressures in Patients with Pulmonary Hypertension: Implications for Clinical Practice. Chest.

[8] Farber HW, Foreman AJ, Miller DP, McGoon MD (2011). REVEAL Registry: Correlation of Right Heart Catheterization and Echocardiography in Patients with Pulmonary Arterial Hypertension. Congest Heart Fail.

[9] Saturni S, Bellini F, Braido F, Paggiaro P, Sanduzzi A, Scichilone N (2014). Randomized Controlled Trials and Real Life Studies. Approaches and Methodologies: A Clinical Point of View. Pulm Pharmacol Ther.

[10] Roland M, Torgerson DJ (1998). What Are Pragmatic Trials?. BMJ.

[11] Lang RM, Badano LP, Mor-Avi V, Afilalo J, Armstrong A, Ernande L (2015). Recommendations for Cardiac Chamber Quantification by Echocardiography in Adults: An Update from the American Society of Echocardiography and the European Association of Cardiovascular Imaging. J Am Soc Echocardiogr.

[12] Rosenkranz S, Preston IR (2015). Right Heart Catheterisation: Best Practice and Pitfalls in Pulmonary Hypertension. Eur Respir Rev.

[13] Bland JM, Altman DG (1986). Statistical Methods for Assessing Agreement between Two Methods of Clinical Measurement. Lancet.

[14] Bland JM, Altman DG (1999). Measuring Agreement in Method Comparison Studies. Stat Methods Med Res.

[15] Vet HC, Terwee CB, Knol DL, Bouter LM (2006). When to Use Agreement versus Reliability Measures. J Clin Epidemiol.

[16] Raymond RJ, Hinderliter AL, Willis PW, Ralph D, Caldwell EJ, Williams W (2002). Echocardiographic Predictors of Adverse Outcomes in Primary Pulmonary Hypertension. J Am Coll Cardiol.

[17] Galiè N, Hoeper MM, Humbert M, Torbicki A, Vachiery JL, Barbera JA (2009). Guidelines for the Diagnosis and Treatment of Pulmonary Hypertension: The Task Force for the Diagnosis and Treatment of Pulmonary Hypertension of the European Society of Cardiology (ESC) and the European Respiratory Society (ERS), Endorsed by the International Society of Heart and Lung Transplantation (ISHLT). Eur Heart J.

[18] Frost A, Badesch D, Gibbs JSR, Gopalan D, Khanna D, Manes A (2019). Diagnosis of Pulmonary Hypertension. Eur Respir J.

[19] Galiè N, Channick RN, Frantz RP, Grünig E, Jing ZC, Moiseeva O (2019). Risk Stratification and Medical Therapy of Pulmonary Arterial Hypertension. Eur Respir J.

[20] Kim NH, Delcroix M, Jais X, Madani MM, Matsubara H, Mayer E (2019). Chronic Thromboembolic Pulmonary Hypertension. Eur Respir J.

[21] Irwig L, Bossuyt P, Glasziou P, Gatsonis C, Lijmer J (2002). Designing Studies to Ensure that Estimates of Test Accuracy are Transferable. BMJ.

[22] Knafl D, Gerges C, King CH, Humbert M, Bustinduy AL (2020). Schistosomiasis-Associated Pulmonary Arterial Hypertension: A Systematic Review. Eur Respir Rev.

[23] Hoeper MM, Humbert M, Souza R, Idrees M, Kawut SM, Sliwa-Hahnle K (2016). A Global View of Pulmonary Hypertension. Lancet Respir Med.

[24] Lapa MS, Ferreira EV, Jardim C, Martins BC, Arakaki JS, Souza R (2006). Clinical Characteristics of Pulmonary Hypertension Patients in Two Reference Centers in the City of São Paulo. Rev Assoc Med Bras.

[25] McGoon MD, Benza RL, Escribano-Subias P, Jiang X, Miller DP, Peacock AJ (2013). Pulmonary Arterial Hypertension: Epidemiology and Registries. J Am Coll Cardiol.

[26] Frost AE, Badesch DB, Barst RJ, Benza RL, Elliott CG, Farber HW (2011). The Changing Picture of Patients with Pulmonary Arterial Hypertension in the United States: How REVEAL Differs from Historic and Non-US Contemporary Registries. Chest.

[27] Yock PG, Popp RL (1984). Noninvasive Estimation of Right Ventricular Systolic Pressure by Doppler Ultrasound in Patients with Tricuspid Regurgitation. Circulation.

[28] Berger M, Haimowitz A, van Tosh A, Berdoff RL, Goldberg E (1985). Quantitative Assessment of Pulmonary Hypertension in Patients with Tricuspid Regurgitation Using Continuous Wave Doppler Ultrasound. J Am Coll Cardiol.

[29] Currie PJ, Seward JB, Chan KL, Fyfe DA, Hagler DJ, Mair DD (1985). Continuous Wave Doppler Determination of Right Ventricular Pressure: A Simultaneous Doppler-Catheterization Study in 127 Patients. J Am Coll Cardiol.

[30] Prada JAV, Ruano J, Martin-Duran R, Larman M, Zueco J, Murua JAO (1987). Noninvasive Determination of Pulmonary Arterial Systolic Pressure by Continuous Wave Doppler. Int J Cardiol.

[31] D’Alto M, Romeo E, Argiento P, D’Andrea A, Vanderpool R, Correra A (2013). Accuracy and Precision of Echocardiography versus Right Heart Catheterization for the Assessment of Pulmonary Hypertension. Int J Cardiol.

[32] Greiner S, Jud A, Aurich M, Hess A, Hilbel T, Hardt S (2014). Reliability of Noninvasive Assessment of Systolic Pulmonary Artery Pressure by Doppler Echocardiography Compared to Right Heart Catheterization: Analysis in a Large Patient Population. J Am Heart Assoc.

[33] Doutreleau S, Canuet M, Enache I, Di Marco P, Lonsdorfer E, Oswald-Mammoser M (2016). Right Heart Hemodynamics in Pulmonary Hypertension - An Echocardiography and Catheterization Study. Circ J.

[34] Venkateshvaran A, Seidova N, Tureli HO, Kjellström B, Lund LH, Tossavainen E (2021). Accuracy of Echocardiographic Estimates of Pulmonary Artery Pressures in Pulmonary Hypertension: Insights from the KARUM Hemodynamic Database. Int J Cardiovasc Imaging.

[35] Zhang RF, Zhou L, Ma GF, Shao FC, Wu XH, Ying KJ (2010). Diagnostic Value of Transthoracic Doppler Echocardiography in Pulmonary Hypertension: A Meta-Analysis. Am J Hypertens.

[36] Taleb M, Khuder S, Tinkel J, Khouri SJ (2013). The Diagnostic Accuracy of Doppler Echocardiography in Assessment of Pulmonary Artery Systolic Pressure: A Meta-Analysis. Echocardiography.

[37] Finkelhor RS, Lewis SA, Pillai D (2015). Limitations and Strengths of Doppler/Echo Pulmonary Artery Systolic Pressure-Right Heart Catheterization Correlations: A Systematic Literature Review. Echocardiography.

[38] Gall H, Yogeswaran A, Fuge J, Sommer N, Grimminger F, Seeger W (2021). Validity of Echocardiographic Tricuspid Regurgitation Gradient to Screen for New Definition of Pulmonary Hypertension. EClinicalMedicine.

