# Supplementary material for: Acurácia do Ecocardiograma Transtorácico como Método de Triagem na Prática Clínica da Investigação da Hipertensão Pulmonar
Source: Arq Bras Cardiol. 2023 Jul 27;120(7):e20220461. [Article in Portuguese] doi: 10.36660/abc.20220461 (PMC10382154; doi:10.36660/abc.20220461)
Supplement: Translation

# Translation: Accuracy of Transthoracic Echocardiogram as a Screening Method in the Clinical Practice of Pulmonary Hypertension Investigation

**Authors:** Camila Farnese Rezende, Eliane Viana Mancuzo, Maria do Carmo Pereira Nunes, Ricardo Amorim Corrêa

**Affiliations:** 1 Brazil Universidade Federal de Minas Gerais – Pós-Graduação Ciências Aplicadas à Saúde do Adulto, Belo Horizonte, MG – Brazil; 2 Brazil Universidade Federal de Minas Gerais – Faculdade de Medicina, Belo Horizonte, MG – Brazil

**Keywords:** Echocardiography, Hypertension, Pulmonary, Data Accuracy

## Abstract

**Background:**

The transthoracic echocardiogram (TTE) plays a screening role in the diagnostic algorithm of pulmonary hypertension (PH). Studies have shown a significant disagreement between TTE measurements of the systolic pulmonary artery pressure (sPAP) and right atrial pressure (RAP) and those obtained by right heart catheterization (RHC).

**Objective:**

To compare TTE measurements of sPAP and RAP with those obtained by RHC in patients being investigated for PH.

**Methods:**

Patients referred to a PH reference center with a high or intermediate TTE probability of PH upon admission were submitted to RHC. The agreement between sPAP and RAP from both procedures was assessed through the Bland-Altman test. Differences of up to 10 mmHg for sPAP and 5 mmHg for RAP were considered within the variability of the test. Receiver Operating Characteristic (ROC) curve was constructed to determine the most accurate sPAP and Tricuspid regurgitation maximal velocity (TRV)values associated with the diagnosis of PH by RHC. The adopted level of statistical significance was 5%.

**Results:**

Ninety-five patients were included. The Bland-Altman analysis showed a bias of 8.03 mmHg (95% CI:-34.9-50.9) for sPAP and -3.30 mmHg (95% CI:-15.9-9.3) for RAP. AUC for sPAP and TRV measured by TTE for discrimination of probable PH were 0.936 (95% CI: 0.836-1.0) and 0.919 (95% CI: 0.837-1.0), respectively. However, only 33.4% of the echocardiographic estimate of sPAP and 55.1% of RAP were accurate, as compared to the measurements obtained by RHC.

**Conclusion:**

TTE has a high discriminatory power as a screening diagnostic method for PH despite presenting disagreements between sPAP and RAP absolute values when compared to RHC measurements.

## Introduction

Pulmonary hypertension (PH) is a broad syndrome currently defined by the presence of a mean pulmonary artery pressure (mPAP) higher than 20 mmHg, established through right heart catheterization (RHC).^1^ PH increases the afterload of the right ventricle (RV), resulting in hypertrophy of the pulmonary artery medial layer, RV dilation with reduction in contractility, which can ultimately lead to right-sided heart failure and death.^2^ PH *per* se is a marker of worse prognosis. PH has been classified into five groups according to the underlying pathogenic mechanisms: Group 1 – Pulmonary arterial hypertension (PAH); Group 2: PH due to left heart diseases; Group 3: PH due to lung diseases and/or hypoxia; Group 4: PH due to pulmonary artery obstructions; Group 5: PH with unclear and/or multifactorial mechanisms.^1 - 4^ PH is further classified as pre-capillary if mPAP is >20mmHg, pulmonary arterial wedge pressure (PAWP) is ⩽15 mmHg and pulmonary vascular resistance (PVR) is >2 WU, encompassing Groups 1, 3, 4, and 5 of the current classification.^4^ A transthoracic echocardiogram (TTE) is indicated in all clinically suspected cases of PH and has a critical role as a screening method for PH diagnosis.^3 , 4^ Tricuspid regurgitation maximal velocity (TRV) and estimated systolic pulmonary artery pressure (sPAP) - obtained through the modified Bernoulli equation where $\mathrm{sPAP}\;=\;4{\mathrm{TRV}}^{2}\;+\;\mathrm{right}\;\mathrm{atrial}\;\mathrm{pressure}\;(\mathrm{RAP})$ - constitute essential parameters for the screening classification as low, intermediate, or high degree of PH probability, the last two being indications for a confirmatory RHC, mostly in suspected cases of PAH.^3 - 5^

However, studies made in the context of research protocols have shown a significant disagreement between the TTE measurement of the sPAP (TTE/sPAP) as compared to that obtained by RHC (RHC/sPAP), due to both underestimation and overestimation of this variable, which may lead to delays in PH diagnosis and clinical management.^6 - 8^ Outside the controlled research environment, in which TTE and RHC are performed blindly and sequentially, in the usual clinical practice of PH diagnosis, the magnitude and direction of such disagreement have not been reported. In this context, observational and pragmatic assessments have been used to evaluate the accuracy of different methods for PH diagnostic evaluation, aiming to achieve more representative data in a non-strictly selected population.^9 , 10^

The purpose of the present study was to assess the agreement between initial TTE/sPAP and RHC/sPAP, TTE/RAP, and RHC right atrial pressure (RHC/RAP) of patients who were referred for diagnostic evaluation at a public PH referral center in Brazil, under the condition of daily clinical practice before RHC and the start of specific PAH drug therapies.

## Methods

### Study population

This study enrolled consecutive patients who had been referred for diagnostic evaluation at the Pulmonary Vascular Disease Department of the Clinical Hospital (Hospital das Clínicas) of the Federal University of Minas Gerais (DVP-HC/UFMG, in Portuguese), in Belo Horizonte, Minas Gerais, from September 2004 to April 2015.

Eligible participants were those who were 18 years of age or older, who had a clinical and laboratory suspicion of Group 1 pulmonary arterial hypertension (PAH) or Group 4 chronic thromboembolic pulmonary hypertension (CTEPH), who had a high or intermediate PH probability according to TTE, and who underwent RHC to confirm diagnosis.^3 , 4^ A maximum 6-month interval between the dates of TTE and the RHC was acceptable in this context.^8^

Patients with a diagnosis of PH due to left heart disease (PH-Group 2), associated with pulmonary diseases and/or hypoxia (PH-Group 3) or with unclear and/or multifactorial mechanisms (PH-Group 5), were deemed ineligible, since RHC is not formally indicated for the diagnosis of these PH groups.^1 , 3 , 4^

This study was approved by the UFMG Ethics Committee (ETIC no. 1.057.219/2015), and all participants who agreed to participate signed the Free and Informed Consent Form.

### Echocardiography and right heart catheterization

TTE and RHC examinations were requested by the attending physicians at the PH Reference Center in accordance with the local protocol and were performed on a regular basis of clinical practice at the cardiology unit of HC/UFMG.

TTE evaluation included the variables peaks of TRV, RAP, and sPAP, the third being estimated by the modified form of Bernoulli’s equation $\left(sPAP=4TRV^{2}+RAP\right)$ . Other TTE parameters suggestive of PH and measures of RV function include the tricuspid annular plane systolic excursion (TAPSE) and RV fractional area change (FAC).^3 - 5 , 11^ RAP was estimated through the inspiratory maneuver as follows: 3mmHg (0-5mmHg) if the diameter of the inferior vena cava (IVC) was smaller than 2.1cm and collapsed more than 50%; 15mmHg (10 – 20mmHg) if the diameter of the IVC was larger than 2.1cm with a collapse of less than 50%. An intermediate value of 8mmHg (5-10mmHg) was used when these criteria were not met.^5 , 11^ Other TTE parameters suggestive of PH were the presence of at least two of the three categories of alterations: a) regarding the ventricles: right ventricle/left ventricle basal diameter ratio>1 and/or flattening of the interventricular septum and TAPSE/sPAP ratio <0.55mm/mmHg, representing a non-invasive measure of RV-pulmonary artery coupling; b) the pulmonary artery: acceleration time in the pulmonary artery <105ms, and/or increased pulmonary regurgitation velocity >2.2m/s, and/or increased diameter of pulmonary artery >25mm); c) IVC and right atrium: IVC larger than 2.1cm associated with its collapse of <50% and/or area of the right atrium >18 cm^2^ .^4 , 5 , 11^

TTE was classified into three categories of pre-RHC probability of PH: a) *high probability* , if the TRV was superior to 3.4m/s or between 2.9 and 3.4m/s if associated with other echocardiographic signs (as above); *intermediate probability* , if TRV was between 2.9 and 3.4m/s with no other signs of TTE, or if TRV was equal or below 2.8m/s associated with at least one additional TTE sign suggestive of PH; and *low probability* if none of these variables were present.^3 , 4 , 11^ These data were obtained from the first TTE performed in the DVP-HC/UFMG, using commercially available equipment (iE33, Epiq 7, Philips Medical Systems, and Aplio 300, Toshiba Ultrasound Systems) and using standardized guidelines.^3 - 5 , 11^ Ecocardiography was performed in routine practice by four different echocardiographers who provide a comprehensive evaluation of RV function and estimation of pulmonary artery pressure. All echocardiographic reports followed a standardized protocol to evaluate patients with pulmonary hypertension.

RHC was performed at the *Cardiovascular Unit of HC-UFMG,* by two professionals with extensive exam experience. Diagnosis of PAH (Group 1) was confirmed if there was an mPAP equal to or higher than 25mmHg at rest and a pulmonary artery wedge pressure (PAWP), left atrium pressure, or left ventricle end-diastolic pressure equal to or less than 15 mmHg (pre-capillary PH) and a pulmonary vascular resistance of 3 Woods Units or more, as defined by the international guidelines in effect at the time of the examinations.^3^ Additionally, PAH patients had to have negative studies for chronic pulmonary thromboembolism (PH Group 4 – a normal or low probability ventilation/perfusion lung scintigraphy or a negative computed tomography pulmonary angiography (CTPA)) and for left-side heart diseases (PH Group 2), lung diseases, or chronic hypoxia (PH Group 3), and PH related to other miscellaneous conditions (PH Group 5). Diagnosis of Group 4 chronic thromboembolic pulmonary hypertension (CTEPH) was established in the presence of a pre-capillary PH (as for PAH) but associated with a high probability ventilation/perfusion lung scintigraphy or a positive CTPA. In the RHC, the RAP, sPAP, mPAP, and PAWP were recorded at the end of a normal expiration. Cardiac output (CO, L/min^1^ ) was calculated based on the indirect Fick method, which estimates the oxygen uptake (VO_2_, ml/min); the cardiac index (CI, L·min^1^·m^2^) was calculated as the ratio of CO to the body surface area. PVR was calculated through the following formula: PVR = (mPAP-PAWP)/CO. All measurements were obtained with reference to the zero level at the mid-thoracic line.^12^

### Statistical analysis

Data distribution was verified through the Shapiro-Wilk test. Descriptive statistics were presented as frequency and percentage, mean (standard deviation) or median (interquartile range), as indicated. The agreements between TTE/sPAP and RHC/sPAP, and TTE/RAP and RHC/RAP, were analyzed using the Bland and Altman method, together with the coefficients of variation (CV) and repetition (CR).^13 - 15^ The estimation of bias (average differences in the measurements of TTE/sPAP and RHC/sPAP, and TTE/RAP and RHC/RAP), its standard deviation (SD) and the 95% limit of agreement were calculated for preparation of the Bland-Altman plot.^13 , 14^ This method evaluates the measurement error, calculated by dividing the standard deviation of the average differences by the square root of two.^15^ The CV is a dispersion measurement that describes the amount of data variability related to the mean, which was calculated using the formula: CV= SD of the average difference of the measurements made by TTE and by RHC *over* the mean of the averages of these measurements multiplied by 100.^14^ The CR express the expected variation of the results for 95% of the repeated measurements, and it is calculated as follows: CR= SD of the average difference of the measurements made by TTE and by RHC multiplied by 1.96.^13^ Differences of 5mmHg for RAP and 10mmHg for sPAP between the TTE and RHC were considered as clinically acceptable.^6^ The Receiver Operating Characteristic (ROC) curve was constructed to determine the most accurate sPAP and TRV values associated with the diagnosis of PH by RHC in the present cohort and to verify the accuracy of TTE/sPAP value > 36mmHg and TRV ≥ 2.80m/s, the formerly recommended cut-off values by the literature to screen symptomatic patients for PH by TTE.^16 , 17^

The sample power was calculated using the paired t-test to allow for the evaluation of the agreement of the sPAP measurements between the TTE and the RHC, using the Minitab Release 14 statistical package. For 80% statistical power in the estimate of the actual difference in the sPAP measurements between the TTE and the RHC, assuming a clinically acceptable difference of 10mmHg and an *alpha* level of 0.05, a sample size of 90 patients was estimated. A p-value of lower than 0.05 was considered significant for all other analyses. The Statistical Package for the Social Sciences, version 18, was used for analyses.

## Results

Ninety-five patients were consecutively admitted in the DVP-HC/UFMG and met the inclusion criteria in the study period. One patient was excluded because it was not possible to retrieve the TTE/sPAP and TTE/RAP measurements preceding the RHC. Five patients had no confirmed diagnosis of PH by RHC. The cohort consisted of middle-aged participants, most of whom were female and in functional class II and III. Approximately two-thirds of the sample consisted of PAH, and the remaining patients had CTEPH. No patient was on PAH drug therapy at the time of the examinations ( Table 1 ).

**Table 1:** – Demographic and clinical characteristics of the study population (n=95)

Characteristics	Data*
**Age, mean ± SD, (years)**	47.6 ± 14.5
**Female, n (%)**	66 (69.4%)
**FC NYHA, n (%)**	
I	8 (8.4%)
II	37 (38.9%)
III	40 (42.1%)
IV	10 (10.5%)
**PH prevalence**	
No PH, n (%)	5 (5.2%)
PH, n (%)	90 (94.8%)
**Interval between noninvasive and invasive measurement, median (IQR), days**	104 (62-153)
**PAH, n (%)**	56 (62.9%)
Schistosomiasis	19 (33.9%)
Idiopathic	14 (25%)
Congenital heart disease	9 (16.1%)
Connective tissue disease	8 (14.8%)
Portopulmonary hypertension	4 (7.1%)
HIV	2 (3.6%)
**CTEPH – n (%)**	33 (37.1%)

*Data are given as mean±SD or median (IQR: interquartile range).CTEPH: chronic thromboembolic pulmonary hypertension; FC: functional class (New York Heart Association); HIV: human immunodeficiency virus; PAH: pulmonary arterial hypertension;PH: pulmonary hypertension.

Data from the TTE and RHC are described in Table 2 .

**Table 2:** – TTE and hemodynamic parameters of the study population (n=95)

Exams	Measured variables	Value
TTE	sPAP, mean ± SD, mmHg*	79.9 ± 24.7
	RAP, mean ± SD, mmHg	12.9 ± 4.7
	TRV, mean ± SD, m/s*	3.78 ± 0.71
	TAPSE, median (IQR), mm	16 (15-18)
	RV-FAC, median (IQR), %	32 (24-38)
	TAPSE/sPAP ratio, median (IQR), mm/mmHg	0.28 (0.19-0.39)
RHC	sPAP, mean ± SD, mmHg	87.6 ± 27.2
	mPAP, mean ± SD, mmHg	70.2 ± 14.4
	RAP, mean ± SD, mmHg	9.6 ± 5.6
	PAWP, median (IQR), mmHg	10.0 (9.2 - 12.4)
	CI, median (IQR), L.min^−1^.m^−2^	2.46 (1.71 - 3.36)
	PVR, median (IQR), Wood units	6.6 (5.1 - 8.2)

Data are given as mean±SD or median (IQR: interquartile range). CI: cardiac index; mPAP: mean pulmonary arterial pressure; PAWP: pulmonary arterial wedge pressure; PVR: pulmonary vascular resistance; sPAP: systolic pulmonary arterial pressure; RAP: right atrial pressure; RHC: right heart catheterization; RV-FAC: right ventricle fractional area change; TAPSE: tricuspid annular plane systolic excursion; TRV: tricuspid regurgitation velocity; TTE: transthoracic echocardiogram. *One patient did not have tricuspid regurgitation and was excluded in the analysis of TTE sPAP and TRV.

A statistically significant *bias* was verified between TTE/sPAP and RHC/sPAP measurements, as shown in Figure 1 and Central Illustration . Regarding the agreement between the parameters ( Table 3 ), the measurement error was of 15.5mmHg, the CV was of 26%, and the CR was of 42.9mmHg.

**Figure 1:** – Bland-Altman plot of systolic pulmonary arterial pressure measured by transthoracic echocardiogram and right heart catheterization. LLA: lower limit of agreement; sPAP: systolic pulmonary arterial pressure; RHC: right heart catheterization; TTE: transthoracic echocardiogram; ULA: upper limit of agreement.

**Central Illustration:** : Accuracy of Transthoracic Echocardiogram as a Screening Method in the Clinical Practice of Pulmonary Hypertension Investigation

Bland-Altman plot of systolic pulmonary arterial pressure and of right atrial pressure measured by transthoracic echocardiogram and right heart catheterization.

**Table 3:** – Accuracy of TTE, sPAP, and TRV to predict pulmonary hypertension*

Variable	Value	Sensitivity	Specificity	PPV	NPV
TTE/sPAP	48 mmHg^1^	90.7%	100%	100%	28.6%
	36 mmHg^2^	97.7%	2.1%	95%	4.6%
TRV	3,08 m/s^1^	90.7%	100%	100%	33.3%
	2,80 m/s^2^	95.3%	5.4%	95%	5.7%

sPAP: systolic pulmonary arterial pressure; TRV: tricuspid regurgitation velocity; TTE: transthoracic echocardiogram; PPV: Positive predictive value; NPV: Negative predictive value. *sPAP measured by right heart catheterization as the reference. For TRV, the reference was the finding of the RHC definition of PH (see the text for definitions). 1- TTE/sPAP and TRV values found in the cohort. 2- TTE/sPAP and TRV values formerly defined in the literature.

Figure 2 and Central Illustration show the agreement between the TTE/RAP and RHC/RAP. A significant bias was found between the measurements.

**Figure 2:** – Bland-Altman plot of right atrial pressure measured by transthoracic echocardiogram and right heart catheterization. LLA: lower limit of agreement; RAP: right atrial pressure; RHC: right heart catheterization; TTE: transthoracic echocardiogram; ULA: upper limit of agreement.

In the present cohort, the higher the values of TTE/sPAP and TRV the greater the discriminatory power for the RHC diagnosis of PH ( Figure 3 and Table 3 ). Conversely, the formerly recommended sPAP and TRV values as the cut-off points of intermediate TTE probability of PAH had higher sensitivities at the expense of lower specificities.^16^

**Figure 3:** – The AUC of the most accurate sPAP and TRV as measured by TTE to predict pulmonary hypertension in the cohort. AUC: area under the curve; NPV: negative predictive value; PPV: positive predictive value; sPAP: systolic pulmonary arterial pressure; TRV: tricuspid regurgitation velocity; TTE: transthoracic echocardiogram.

By the adopted definition of accuracy for sPAP (variation up to 10mmHg) and RAP (variation up to 5mmHg) in TTE as compared to those of RHC, the estimates of RAP and sPAP were poor ( Figure 4 ). TTE underestimated the sPAP values by 41.5% *versus* 25.1% (-30.4±10.2 *versus* 15.2±8.9mmHg; p=0.04) and overestimated the RAP values by 33.7% *versus* 11.2% (11.3±4.8 *versus* -8.4±3.7mmHg; p=0.03) in cases in which a difference was above the pre-defined acceptable variation. An illustration of TTE evaluation of estimated sPAP and RAP in a patient with Schistosomiasis associated with PAH and the difference of these measurements in the RHC are provided in Figure 5 .

**Figure 4:** – Differences clinically acceptable for sPAP and RAP between the transthoracic echocardiogram and right heart catheterization. sPAP: systolic pulmonary arterial pressure; RAP: right atrial pressure. *Differences of 5mmHg for RAP and 10mmHg for sPAP between the transthoracic echocardiogram and the right heart catheterization were considered to be clinically acceptable.

**Figure 5:** – Disagreement between systolic pulmonary artery pressure and right atrial pressure measured through TTE and RHC in a patient with Schistosomiasis-associated pulmonary hypertension. IVC: inferior vena cava; IVS: interventricular septum; RA: right atrium; RHC: right heart catheterization; RV: right ventricle; sPAP: systolic pulmonary arterial pressure; TTE: transthoracic echocardiogram.

## Discussion

The present single-center study sought to evaluate the accuracy of TTE in the screening of PH in patients referred to a PH reference center in the context of daily clinical practice. TTE plays a pivotal role in the clinical scenario of the diagnosis of PH.^3 , 4^ With clinical and laboratory data, TTE probability of PH directs the investigation to one of the current five groups of the disease, which requires other specific procedures and therapies.^3 , 4 , 18^ Patients who have an intermediate or high TTE probability of PH and are presumed to have PAH (PH Group 1) or CTEPH (PH Group4) are primarily referred for invasive diagnosis through RHC as they are candidates for using specific therapeutic modalities, such as PAH drug therapy or combined pulmonary thromboendarterectomy, and/or pulmonary angioplasty, and/or drug CTEPH therapy.^19 , 20^

The present study demonstrates that both TTE/sPAP of more than 36 mmHg and TRV of 2.80m/s or more have high sensitivities in the screening of PH in symptomatic patients, which is a desirable performance when a test is used to screen the diagnosis of severe diseases.^21^

The sample enrolled in this study encompassed all patients referred to the DVP-HC/UFMG that were consecutively admitted during the study period and consisted of a larger number of PAH (62.9%) than those with CTEPH (37.1%). Due to referral *bias* , the number of patients whose RHC was negative for PH was very small (5.2%). Unlike reports from northern countries, *Schistosomiasis mansoni* is our most prevalent PAH etiology due to the endemicity of this infection, particularly in our region.^22 - 24^ However, the average age and sex distribution were similar to reports from countries in the northern hemisphere, demonstrating the impact of the disease in middle-aged individuals even in diverse epidemiological contexts.^25 , 26^ Likewise, most of the patients, upon admission, were in FC III and IV (52.6%). Unfortunately, the delay in PH diagnosis is a global issue that contributes to the worsening of the disease to advanced stages, including right heart failure and a high risk of death.^25 , 26^

### Comparison between echocardiographic and invasive hemodynamic measurements of right heart chamber pressures

The accuracy of the TTE in providing the diagnosis of PH has been evaluated since the 1980’s. TTE is currently a relatively low cost, largely available, and non-invasive procedure. Yock and Popp reported a good correlation between TTE/sPAP and RHC/sPAP in 54 patients (r=0.93, SEE = 8mmHg).^27^

However, there is some discussion about the appropriateness of using common correlation tests when two distinct methods evaluate the same quantitative dependent variable.^13^ The Bland-Altman method is considered more appropriate for this purpose. Findings from Fisher et al. showed a significant *bias* of the TTE in sPAP estimation (-0.6mmHg; 95% limit of agreement: -40.0 to 38.8mmHg) in 65 patients who underwent TTE and RHC in a blinded design and with a one-hour interval between the tests.^6^ Two other studies also reported a *bias* that ranged from 2.2 to 8mmHg in the TTE estimation of sPAP (95% limit of agreement: -34.2 to 38.6mmHg and -28.4 to 44.4mmHg).^7 , 8^ The analysis of the REVEAL registry data showed a low accuracy of the pre-RHC TTE in 57.4% of sPAP estimates (> 10mmHg higher or lower than the RHC measure) and in 36.5% of RAP estimates (>5mmHg higher or lower than RAP in RHC) in examinations made within a larger interval than ours, a maximum 12-month interval between the exams.^8^

However, other authors have demonstrated a better agreement between measurements of sPAP, but the level of agreement in the difference in the mean was larger, indicating only a moderate precision of the echocardiographic measurements.^28 - 30^ Although D’Alto M et al. found no significant bias (-0,5mmHg) between TTE/sPAP and RHC/sPAP with broad limits of agreement (-19mmHg to 18mmHg), in contrast to other reports, RHC was recommended for reasons other than PAH (PAH: 36%; pulmonary venous hypertension: 40%; lung disease PH:16%).^31^ A large retrospective study (n=1695) enrolled patients who had a maximum of five days of interval between TTE and RHC. The main indications were left heart disease (59%), valve disease (27%), and PAH (6%). They found a mean sPAP of 45.3 ± 15.5mmHg by TTE and 47.4 ± 16.4mmHg by RHC, showing a strong correlation between measurements of sPAP(r = 0.87; p<0.0001) and RAP (r = 0.82; p<0.0001).^32^ Through the Bland-Altman analysis, there were a -2mmHg *bias* for sPAP (95% limit of agreement: -18.1 to 14.1mmHg) and a 1mmHg *bias* for RAP (95% limit of agreement: 0.1 to 1.9mmHg).^32^ Doutreleau et al. compared TTE and RHC, which were sequentially performed in 106 patients with suspected or confirmed PH (mean delay, 16 min). PH was not confirmed in 16.9% of the patients, 10.4% were diagnosed with post-capillary PH, and 72.7% with pre-capillary PH. The correlations were strong (for sPAP: r=0.84; for RAP: r=0.70), and the Bland-Altman analysis showed a significant *bias* of 1.4mmHg for sPAP (95% limit of agreement: -22.6 to 25.4mmHg) and 1.9mmHg for RAP (95% limnit of agreement: -6.1 to 9.9mmHg).^33^ Other authors evaluated consecutive patients and recommended RHC (PAH and CTEPH: 40%; heart failure: 42%), using a larger interval of up to three hours between the tests, and reported a minimal *bias* (mean bias = +2.4mmHg) between the TTE and invasive sPAP measures but with a broad limit of agreement (-20 to +25mmHg).^34^

Three meta-analysis and one systematic review evaluated the accuracy of the estimation of sPAP by TTE, but, overall, the results were inconsistent due to a significant heterogeneity among the studies regarding the inclusion of participants without disease and others with left and right heart disease. Moreover, most studies employed the usual correlation tests, which are less appropriate as agreement measurements between tests that measure the same quantitative variable.^35 - 37^

Recently published international guidelines have updated the hemodynamic definition of PH by lowering the mPAP value from ≥ 25 mmHg to > 20 mmHg.^4^ This adjustment was based on the previously reported values found in non-PH individuals and on the increasing body of evidence of the prognostic impact of even mild elevations of mPAP (between 20 and 24 mmHg) in some PAH subgroups.^4^ Nevertheless, TTE remains the most appropriate screening method for PH diagnosis. In this new scenario, Gall and colleagues evaluated a large retrospective sample of PH-confirmed patients and found that the tricuspid regurgitation gradient meets the new definition PH criteria and that decreasing the lower limit of TRV does not improve the screening yield of TTE.^38^

To the best of our knowledge, this is the first Brazilian published study that compared TTE and RHC pressures in adult patients with a suspected diagnosis of PAH and CTEPH in the context of daily clinical practice. The present study showed a high discriminatory power of the TTE/sPAP and TRV for the diagnosis of PH. According to our definitions (difference of 10mmHg for sPAP and 5mmHg for RAP), only 33.4% of the TTE/sPAP and 55.1% of TTE/RAP estimations were accurate, which was similar to the results found by Fisher et al.^6^ (sPAP: 52%), Rich et al.^7^ (sPAP: 49.4%), and REVEAL analysis^8^ (sPAP: 42.6% and RAP: 63.5%). Doutreleau et al.^33^ and Venkateshvaran et al.^34^ found somewhat more reliable measurements of sPAP (68% and 62%, respectively) and RAP in 79% of the patients.^34^ Regarding the direction of the disagreements, the present study showed that TTE underestimated the sPAP and overestimated the RAP with higher frequency (sPAP: 41.5% and 25.1%; RAP: 33.7% and 11.2%) and in the magnitude of the absolute differences (sPAP:-30.4±10.2 versus 15.2±8.9mmHg; RAP:11.3±4.8 versus -8.4±3.7mmHg), all higher than the pre-defined acceptable differences of 10mmHg and 5mmHg, respectively. Fisher et al. found that sPAP values were more underestimated than overestimated (-30±16 versus19±11mmHg; p=0.03).^6^ Rich et al.^7^ and Faber et al^8^ also found similar data using the REVEAL registry (sPAP: underestimated in 30% and 20.6% in the TTE, and in 34.8% and 22.5%, respectively; RAP: overestimated in 26.3% versus 12.4%).^8^ This scenario illustrates how difficult it is to ascertain the severity of PH and to stratify its risk using only TTE.

Some limitations of this study should be mentioned. First, the time elapsed between TTE and RHC may raise some concern. However, due to the observational design and the objectives of this study, TTE and RHC could not be performed in a short interval period. Moreover, we found a 3.3-month average interval, which is acceptable when suspected PH patients are referred by non-specialists for evaluations at PH reference centers within the context of clinical practice in a public health environment. Reports from the Reveal Registry - a landmark PH registry – analyzed this issue in even more larger intervals.^7 , 8^ Second, some variability between examiners might be expected, since the TTE that raised the suspicion of PH was performed by different examiners. However, all of tests were performed in the same cardiologic unit of HC/UFMG and were regularly scheduled in the context of usual medical care. Conversely, this study had the very purpose of evaluating the consistency of the TTE as a screening for PH out of the controlled research environment. In in this sense, we expected even larger disagreements than those reported in the literature. In this regard, we consider the present results to be highly consistent, since they reproduce previous reports and reflect, at least in part, inherent limitations of the methods, regardless of the geographic contexts in which they are performed.^5 , 11 , 37^

## Conclusion

In conclusion, TTE plays a key role in the screening evaluation of PH-suspected patients and has a high discriminatory power even in the context of usual clinical practice. The disagreements between sPAP and RAP measurements reinforce TTE as a valid screening tool and the need to perform RHC in the context in which a definitive PH diagnosis is recommended.
